# Uterine Fibroids Increase the Risk of Thyroid Cancer

**DOI:** 10.3390/ijerph17113821

**Published:** 2020-05-28

**Authors:** Li-Min Sun, Li-Min Chung, Cheng-Li Lin, Chia-Hung Kao

**Affiliations:** 1 Department of Radiation Oncology, Zuoying Branch of Kaohsiung Armed Forces General Hospital, Kaohsiung 81342, Taiwan; limin.sun@yahoo.com; 2 Institute of Medical Science and Technology, National Sun Yat-sen University, Kaohsiung 80424, Taiwan; 3 Department of Medical Oncology, Zuoying Branch of Kaohsiung Armed Forces General Hospital, Kaohsiung 81342, Taiwan; asama0702@gmail.com; 4 Management Office for Health Data, China Medical University Hospital, Taichung 404, Taiwan; orangechengli@gmail.com; 5 College of Medicine, China Medical University, Taichung 404, Taiwan; 6 Graduate Institute of Biomedical Sciences, College of Medicine, China Medical University, Taichung 404, Taiwan; 7 Department of Nuclear Medicine and PET Center, China Medical University Hospital, Taichung 404, Taiwan; 8 Department of Bioinformatics and Medical Engineering, Asia University, Taichung 413, Taiwan; 9 Center of Augmented Intelligence in Healthcare, China Medical University Hospital, Taichung 404, Taiwan

**Keywords:** uterine fibroids, thyroid cancer, population-based cohort study

## Abstract

Objectives: The formation of uterine fibroids (UF) is potentially linked to the development of thyroid cancer through a common factor: female sex hormones. Methods: We conducted a nationwide population-based cohort study to determine whether Taiwanese women with UF have an increased risk of thyroid cancer. The data of both the UF and control groups were derived from the National Health Insurance Research Database (NHIRD) of Taiwan. Groups were matched by the year of UF diagnosis, age, income, urbanization level, occupation, and comorbidities. A Cox proportional hazard regression model was used to compare the incidence of thyroid cancer between the UF and control groups. In addition, the model was used to determine the hazard ratio of thyroid cancer in the UF group in comparison with the control group. Results: Women with UF had a statistically significantly increased risk of thyroid cancer compared with controls (adjusted hazard ratio (aHR): 1.64, 95% confidence interval (CI): 1.26–2.13). Stratified analyses showed that women with UF who had a significantly increased risk of thyroid cancer were more likely to be middle aged, have middle and higher income levels, and a medium follow-up period (1–5 years) of UF. No other UF patient characteristics and comorbidities showed association with the risk of thyroid cancer. In addition, UF patients had a significantly increased risk of thyroid cancer regardless of whether or not they underwent myomectomy. Conclusions: The results suggest that women with UF have an increased risk of subsequent thyroid cancer. Further research is needed to explore whether surveillance strategies for the early detection of thyroid cancer using ultrasonography should be implemented among patients with UF.

## 1. Introduction

Uterine fibroids (UF) are the most common benign tumor in women. However, the majority of patients do not have any symptoms, and their UF are often detected incidentally during routine health examinations [1]. UF are also the main reason for women undergoing hysterectomy [2]. Statistics show that women of reproductive age have a more than 70% chance of developing UF, and the incidence of UF in women decreases dramatically after menopause [3]. Therefore, it is speculated that the development of UF is related to female hormones, which is supported by the results of earlier studies [4,5].

Thyroid cancer is the most common endocrine cancer, with an incidence that is increasing each year worldwide [6]. Advances in diagnostic imaging tools have led to an increasing number of cases being diagnosed early, and this may also be related to the increase in the true incidence [6]. According to cancer statistics for 2016, thyroid cancer is the fourth most common cancer among females in Taiwan [7]. Thyroid cancer is one of the few cancers in which the incidence rate is higher in women than in men. Worldwide data show that women are two to four times more likely to develop thyroid cancer than men; therefore, female hormones may play important roles in its etiology [8]. In the United States, from 2006 to 2010, the average annual percentage increase in thyroid cancer among men and women was 5.4% and 6.5%, respectively [9]. In Taiwan, from 1997 to 2016, the average annual percentage increase among men and women was 6.9% and 4.6%, respectively [10]. Differentiated carcinoma (papillary and follicular types), medullary carcinoma, and anaplastic carcinoma are the main types of thyroid cancer. Papillary carcinoma comprises about 85% of all thyroid cancers, and the 10-year disease-specific mortality rate for differentiated thyroid carcinoma is less than 5% [11]. Compared with most malignancies, the prognosis for thyroid cancer is relatively good, but it is a topic worthy of attention in public health research because it occurs most frequently in younger age groups [12], which have relatively higher productivity.

Because the occurrence of both UF and thyroid cancer may be related to female hormones, some studies have explored the link between the two diseases and have found that women with a history of UF had a significantly increased risk of thyroid cancer [8,13]. Moreover, researchers also investigated the relationship between hysterectomy and thyroid with inconclusive results [13,14,15]. Through a nationwide database, we designed this study to determine whether Taiwanese women with UF have an increased risk of subsequent thyroid cancer and whether the history of myomectomy affects this possible relationship.

## 2. Methods

### 2.1. Data Source

The single-payer and compulsory National Health Insurance (NHI) program, which has been operational since 1995, covers approximately 99% of all 23 million Taiwanese residents. The National Health Research Institutes (NHRI) receive data regarding insurance claims from the National Health Insurance Administration (NHIA), which are subsequently compiled into the National Health Insurance Research Database (NHIRD) for research purposes. For this retrospective cohort study, we obtained data from the Longitudinal Health Insurance Database (LHID2000), a subset of NHIRD that comprises the medical records of one million randomly sampled beneficiaries sampled from the NHIRD.

The database included the disease history, based on the International Classification of Diseases, Ninth Revision, Clinical Modification (ICD-9-CM), of all insured inpatients and outpatients. To protect privacy, all personal information is scrambled into surrogate identification codes before being released to the public. This study was approved by the Research Ethics Committee of China Medical University Hospital (CMUH104-REC2-115-CR-4).

### 2.2. Sampled Participants

The UF cohort included female patients aged ≥20 years who had received a new diagnosis of UF (ICD-9-CM code 218) between 1 January 2000, and 31 December 2012. The initial date of the UF diagnosis was defined as the index date. For each UF patient, one comparison subject without UF was frequency matched by age group (5-year intervals), monthly insured income, occupation, urbanization level, and comorbidities [Hashimoto’s thyroiditis (ICD-9-CM code 245.2), goiter (ICD-9-CM codes 240, 240.9, 241.0, 241.1, 241.9, 242.0, 242.1, 242.2, 242.3, and 246.9), diabetes (ICD-9-CM codes 250 and A181), hypertension (ICD-9-CM codes 401–405), hyperlipidemia (ICD-9-CM code 272), alcohol-related illness (ICD-9-CM codes 291, 303, 305, 571.0, 571.1, 571.2, 571.3, 790.3, A215, and V11.3)], chronic obstructive pulmonary disease (COPD) (ICD-9-CM codes 491, 492, and 496), and the year of UF diagnosis. Participants in both cohorts with a history of thyroid cancer before the index date or those aged under 20 years were excluded.

Thyroid cancer (ICD-9-CM code 193) was defined as the endpoint of this study. All the participants were followed up from the index date to the occurrence of thyroid cancer, withdrawal from the insurance program, or the end of 2013, whichever occurred first.

### 2.3. Statistical Analysis

The distributions of the sociodemographic data and comorbidities were compared between the UF cohort and the control cohort using the chi-square test for categorical variables and the t test for continuous variables. The Kaplan–Meier method was used to estimate the cumulative incidence of thyroid cancer in both cohorts, with significance based on the log-rank test. The incidence rate of thyroid cancer for each cohort was calculated as the total number of thyroid cancer events divided by 10,000 person-years. Univariable and multivariable Cox proportional hazards regression analyses were used to estimate the hazard ratios (HRs) and 95% confidence intervals (CIs) for thyroid cancer incidence in the UF cohort relative to the comparison cohort. The risks of thyroid cancer were analyzed by stratifying the study population according to age (≤34 years, 35–49 years, and ≥50 years), insured monthly income (NT$ < 15,000, 15,000–30,000, and >30,000), urbanization level (four levels, with 1 being the most urbanized and 4 being the least), occupation (office worker, laborer, and other), comorbidities, and follow-up period (<1 year, 1–5 years, and ≥5 years). We further examined the relationship between thyroid cancer and myomectomy among the UF cohort. SAS software version 9.4 (SAS Institute Inc, Cary, NC, USA) was used to conduct all statistical analyses.

## 3. Results

The demographic characteristics and comorbidities of the UF and control cohorts are shown in Table 1. After frequency matching, 57,066 patients with UF and 57,066 controls without UF were analyzed. Almost all of the characteristics were found to be similar between the two cohorts. The mean ages of the UF and control cohorts were 42.2 ± 8.76 and 41.9 ± 9.40 years, respectively, with the most common age group being 35–49 years. The UF cohort had a higher prevalence of goiter and COPD at baseline compared with the control cohort (*p* < 0.05). No significant differences were observed in the prevalence rates of other demographic characteristics and comorbidities between the two cohorts (*p* > 0.05).

**Figure 1:**
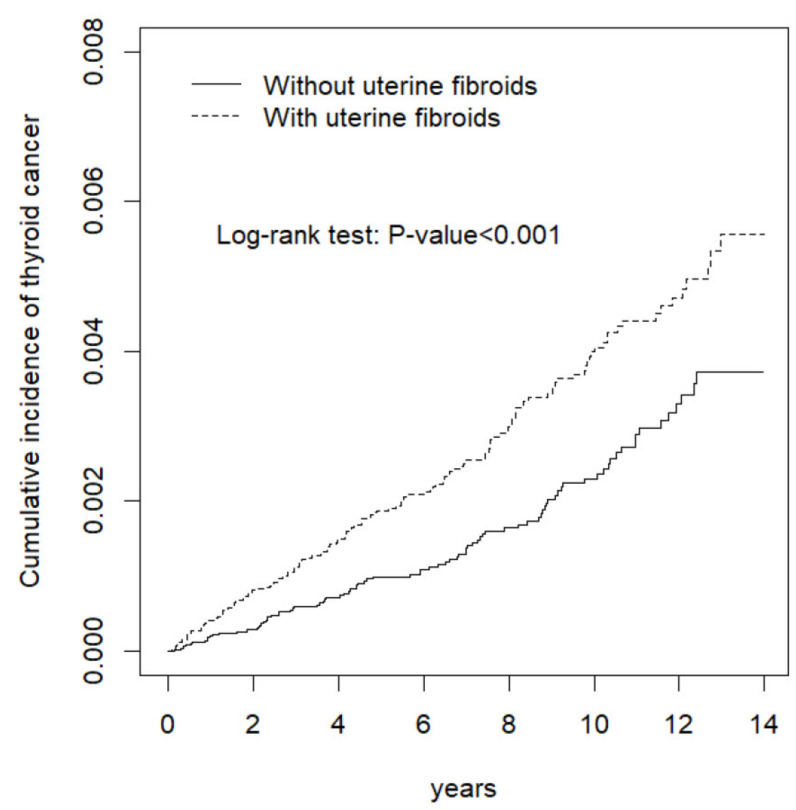
Cumulative incidence of thyroid cancer between women with and without uterine fibroids.

The cumulative incidence of thyroid cancer in the UF cohort was higher than that in the control cohort in Kaplan–Meier analysis with a log-rank test (*p* < 0.001; Figure 1). Table 2 compares the person-years, incidence rate, and HR for the risk of thyroid cancer between the UF and control cohorts; the analyses were stratified according to several variables, namely, age, insured monthly income, occupation, urbanization level, comorbidity status, and follow-up duration. The overall incidence of thyroid cancer was higher in the UF cohort than in the control cohort (3.90 vs. 2.36 per 10,000 person-years). Multivariate analysis using the Cox regression model showed that the UF cohort had a significantly increased risk of thyroid cancer, with an aHR of 1.64 (95% CI: 1.26–2.13). Compared with the control cohort, the age-specific risk of thyroid cancer for the UF cohort was significantly higher in patients aged 35–49 years, with an aHR of 1.67 (95% CI: 1.22–2.30). This result indicates that UF remained significantly associated with a higher risk of thyroid cancer in the following subgroups: patients with insured monthly income NTD 15,000–30,000 and >30,000; patients living regions with urbanization levels 1, 2, and 4; patients who were office workers or laborers; patients with or without comorbidities; and patients with 1–5 years of follow-up. 

Compared with controls, UF patients had a significantly increased risk of thyroid cancer regardless of whether or not they underwent myomectomy; however, after adjusting for all variables, no significant association was found between thyroid cancer and myomectomy among UF women, with an aHR of 1.06 (95% CI: 0.74–1.52) (Table 3). 

## 4. Discussion

This population-based study indicates that Taiwanese women with UF have a significantly increased risk of subsequent thyroid cancer when compared with controls. Further stratified analyses revealed that the increased risk of thyroid cancer was most prevalent in middle-age women, women with middle and higher incomes, and women with a medium UF follow-up period (1–5 years). Other factors of patients’ characteristics and comorbidities did not show any specific tendency for the risk of thyroid cancer. Moreover, myomectomy status had no effect on the risk of thyroid cancer among UF women.

Both the uterus and thyroid are hormone responsive organs; therefore, it is plausible that the mechanisms linking UF and thyroid cancer may be related to female sex hormones. UF are most prevalent during reproductive years and usually regress after menopause. UF seem to be hypersensitive to female steroid hormones, distinct from the normal myometrial response to estrogen and progesterone. At the tumoral level, the expression of α-estrogen (ER-α) and progesterone (PR) receptors was found to be higher on the myometrial surface [16,17]. The incidence of thyroid cancer is 2–4 times higher in reproductive-age women compared with men [18]; thus, estrogen is a possible risk factor [19,20]. Estrogen receptors have been found in both normal and neoplastic thyroid tissue [21]. Faria et al. reviewed the literature and found evidence demonstrating that estrogen induces cell growth in primary cultures of human thyrocytes obtained from benign and malignant thyroid nodules and in most human thyroid carcinoma cell lines [22,23,24]. In addition to hormone effects, UF and thyroid cancer may share another risk factor: obesity [4,6,10,16,17,25,26,27,28]. An earlier study conducted by Lumbiganon et al. highlighted a 6% increase in UF risk for each BMI unit increase [26], and Ross et al. found that for every 10 kg increase in body weight, there was more than a 20% increase of UF [27]. In addition, a pooled analysis of five prospective studies indicated that obesity was positively associated with thyroid cancer risk in both men and women in the United States [28].

Guenego et al. found that women with a history of UF, irrespective of whether they had a hysterectomy or not, were at a higher risk of thyroid cancer (HR: 1.91; 95% CI: 1.50–2.44). When they considered history of UF and hysterectomy simultaneously, each variable remained statistically associated with the risk of thyroid cancer in the same multivariate statistical model [hysterectomy (yes/no): HR: 1.70, 95% CI: 1.27–2.28; UF (yes/no): HR: 1.33, 95% CI: 1.01–1.75] [13]. Luo et al., indicated that women who had undergone a hysterectomy had a significantly increased risk of thyroid cancer when compared with women who had not undergone a hysterectomy (HR: 1.46, 95% CI: 1.16–1.85) [14]. One case-control population-based study detected an increased risk of thyroid cancer among women with a history of hysterectomy, although it was borderline nonsignificant [29]. To determine whether UF-specific surgery affects the risk of thyroid cancer among UF patients, we investigated the effect of myomectomy on the risk of thyroid cancer among UF patients. Compared with controls, we found a significant increase in the risk of thyroid cancer in patients with and without myomectomy; however, when we restricted patients with UF, there was no significant association between thyroid cancer and myomectomy (Table 3). 

For identifying comorbidities that may be related to the risk of either UF or thyroid cancer, we collected available information from the NHIRD. The increasing incidence of Hashimoto’s thyroiditis in the past two decades paralleled the trend of increased incidence of thyroid cancer, although the link between them remains debatable [25,30]. Mack et al. discovered that goiter is an independent risk factor for thyroid cancer in female patients in Los Angeles county [15]. We used alcohol-related illness and COPD as the surrogates for alcohol and cigarette exposure, respectively.

One of the main advantages of this study, conducted using a large national database, is its representativeness of the Taiwanese population. However, this research has some limitations. First, the histology type of thyroid cancer cannot be identified from the NHIRD. Braganza et al. found that UF was associated with an increased risk of thyroid cancer, and the results were similar when the outcome was restricted to papillary carcinoma; however, the risk became stronger after the outcome was restricted to follicular thyroid carcinoma (HR: 2.75, 95% CI: 1.13–6.68) [8]. The current study could not verify similar findings. Second, patients’ lifestyle records are unavailable in the NHIRD; some important factors, such as family history of UF or thyroid cancer, BMI, or body weight, cannot be adjusted for in the analyses. Third, surveillance bias may exist because patients with UF might have more opportunities to visit doctors; consequently, there is an increased chance of related thyroid examinations to be performed. In addition, research has shown that patients with UF have an increased risk of thyroid nodules [31,32], and patients with thyroid nodules undergo thyroid examinations, increasing their chance of finding thyroid cancer as a result. However, surveillance biases are less likely to occur in our study because the increased risk of thyroid cancer was noted in the UF patients with 1–5 years of follow-up and not in those with <1 year of follow-up.

## 5. Conclusions

This population-based study determined a significantly increased risk of thyroid cancer among Taiwanese women with UF. Female sex hormones may underlie the association between UF and thyroid cancer. Given the relatively good outcome of younger women with thyroid cancer, thyroid cancer surveillance strategies should be modified to include patients with UF, enabling the early detection of curable diseases using modern diagnostic imaging tools. Nevertheless, additional comprehensive studies are warranted to clarify this issue.

## Figures and Tables

**Table 1:** Characteristics of demography and comorbidities distribution for patients with and without uterine fibroids.

	Uterine Fibroids		*p*-Value
	No	Yes	
	(*N* = 57,066)	(*N* = 57,066)	
Age stratified			0.34
≤34	10,577 (20.8)	10,613 (20.9)	
35–49	32,102 (63.2)	32,237 (69.5)	
50+	8087 (15.9)	7916 (15.6)	
Age, mean ± SD ^a^	41.9 ± 9.40	42.2 ± 8.76	<0.001
Monthly insured income (NTD) ^†^			0.99
<15,000	16,230 (32.0)	16,223 (32.0)	
15,000–30,000	17,549 (34.6)	17,538 (34.6)	
>30,000	16,987 (33.5)	17,005 (33.5)	
Urbanization level ^‡^			
1 (highest)	18,249 (36.0)	18,215 (35.9)	
2	15,210 (30.0)	15,202 (30.0)	
3	8434 (16.6)	8448 (16.6)	
4 (lowest)	8873 (17.5)	8901 (17.5)	
Occupation category			0.92
Office worker	30,225 (59.6)	30,196 (59.5)	
Laborer	17,096 (33.7)	17,132 (33.8)	
Other ^&^	3415 (6.73)	3438 (6.77)	
Comorbidity			
Hashimoto’s thyroditis	95 (0.19)	94 (0.19)	0.94
Goiter	3202 (6.31)	3369 (6.64)	0.03
Hypertension	6889 (13.6)	7039 (13.9)	0.17
Diabetes	797 (1.57)	873 (1.72)	0.06
Hyperlipidemia	7006 (13.8)	7153 (14.1)	0.18
COPD	2971 (5.85)	3140 (6.19)	0.03
Alcohol-related illness	679 (1.34)	703 (1.38)	0.52

Chi-square test; ^a^ *t* test; ^†^ New Taiwan Dollar (NTD), 30 NTD is equal to 1 USD. ^‡^ The urbanization level was divided into four levels based on the population density of the residential area, with level 1 being the most urbanized and level 4 being the least urbanized. ^&^ Other occupation categories included those for women who were primarily retired, unemployed, and in low-income populations.

**Table 2:** Incidences and hazard ratios of thyroid cancer between women with and without uterine fibroids, stratified by demographics, comorbidities, and follow-up period.

	Uterine Fibroids							
	No			Yes				
Variable	Event	PY	Rate ^#^	Event	PY	Rate ^#^	Crude HR (95% CI)	Adjusted HR ^$^ (95% CI)
All	89	377,567	2.36	148	379,170	3.90	1.65 (1.27, 2.15) ***	1.64 (1.26, 2.13) ***
Age group, years								
≤34	16	75,278	2.13	19	76,688	2.48	1.16 (0.60, 2.26)	1.12 (0.58, 2.19)
35–49	60	244,733	2.45	102	246,238	4.14	1.69 (1.23, 2.32) **	1.67 (1.22, 2.30) **
50+	13	57,555	2.26	27	56,245	4.80	2.12 (1.10, 4.12) *	1.93 (0.99, 3.76)
Insured monthly income (NTD) ^†^								
<15,000	25	107,172	2.33	30	107,592	2.79	1.19 (0.70, 2.03)	1.18 (0.69, 2.01)
15,000–30,000	28	135,197	2.07	52	136,471	3.81	1.84 (1.16, 2.91) **	1.81 (1.15, 2.87) *
>30,000	36	135,197	2.66	66	135,108	4.88	1.83 (1.22, 2.75) **	1.82 (1.21, 2.73) **
Urbanization level ^‡^								
1 (highest)	32	137,981	2.32	51	138,141	3.69	1.59 (1.02, 2.48) *	1.59 (1.02, 2.47) *
2	30	113,164	2.65	49	113,672	4.31	1.62 (1.03, 2.56) *	1.60 (1.01, 2.51) *
3	16	61,871	2.59	15	62,703	2.39	0.92 (0.46, 1.87)	0.91 (0.45, 1.84)
4 (lowest)	11	64,551	2.59	33	64,653	5.10	3.00 (1.52, 5.93) **	2.96 (1.49, 5.85) **
Occupation category								
Office worker	54	223,429	2.42	80	224,066	3.57	1.48 (1.05, 2.08) *	1.46 (1.04, 2.06) *
Laborer	30	130,080	2.31	58	130,767	4.44	1.92 (1.24, 2.99) **	1.90 (1.22, 2.95) **
Other ^&^	5	24,058	2.08	10	24,337	4.11	1.98 (0.68, 5.78)	1.94 (0.66, 5.68)
Comorbidity								
No	52	266,441	1.95	84	266,508	3.15	1.61 (1.14, 2.28) **	1.61 (1.14, 2.27) **
Yes	37	111,126	3.33	64	112,662	5.68	1.71 (1.14, 2.56) **	1.70 (1.14, 2.55) *
Follow-up period								
<1 year	10	50,599	1.98	21	50,618	4.15	2.10 (0.99, 4.46)	2.04 (0.96, 4.33)
1–5 years	33	169,545	1.95	62	169,907	3.65	1.87 (1.23, 2.86) **	1.84 (1.21, 2.81) **
≧5 years	46	157,423	2.92	65	158,645	4.10	1.40 (0.96, 2.04)	1.40 (0.96, 2.04)

Rate ^#^, incidence rate, per 10,000 person-years; crude HR, relative hazard ratio. ^†^ New Taiwan Dollar (NTD), 30 NTD is equal to 1 USD. ^‡^ The urbanization level was divided into four levels according to the population density of the residential area. Level 1 represented the most urbanized and level 4 represented the least urbanized. ^&^ Other occupation categories included those for women who were in low-income populations, retired, and unemployed. Adjusted HR ^$^: multivariable analysis by age, occupation, urbanization, insured monthly income (NTD), and comorbidities. * *p* < 0.05, ** *p* < 0.01, *** *p* < 0.001.

**Table 3:** Risk of thyroid cancer related to myomectomy in patients with uterine fibroids.

Variable	Event	PY	Rate ^#^	Adjusted HR (95% CI)	Adjusted HR ^†^ (95% CI)
Control patients	89	377,567	2.36	1.00	
Uterine fibroids patients					
Myomectomy					
No	106	275,774	3.84	1.61 (1.21, 2.13) **	1.00
Yes	42	103,397	4.06	1.71 (1.19, 2.47) **	1.06 (0.74, 1.52)

Rate ^#^, incidence rate, per 10000 person-years. Adjusted HR ^†^: multivariable analysis by age, occupation, urbanization, insured monthly income (NTD), and comorbidities. ** *p* < 0.01.

## List of Abbreviations

UF: uterine fibroids; NHIRD: National Health Insurance Research Database; aHR: adjusted hazard ratio; CI: confidence interval; LHID2000: Longitudinal Health Insurance Research Database 2000; ICD-9-CM: International Classification of Diseases, Ninth Edition, Clinical Modification.
